# Treatment outcome of multidrug-resistant tuberculosis in Japan – the first cross-sectional study of Japan tuberculosis surveillance data

**DOI:** 10.1186/s12879-018-3353-9

**Published:** 2018-08-31

**Authors:** Lisa Kawatsu, Kazuhiro Uchimura, Kiyohiko Izumi, Akihiro Ohkado, Takashi Yoshiyama

**Affiliations:** Department of Epidemiology and Clinical Research, the Research Institute of Tuberculosis, 3-1-24, Matsuyama Kiyose, Tokyo, Japan

**Keywords:** Tuberculosis, Multidrug-resistance, Treatment outcome, Epidemiology, Surveillance

## Abstract

**Background:**

Multidrug resistant-tuberculosis (MDR-TB) is a major global health concern. Its treatment requires toxic medications, is longer and costlier than drug-susceptible TB, and often results in productivity losses and poor outcomes. In Japan, a TB middle-burden country, reports on treatment outcome of MDR-TB patients have only been institution-based. We thus sought to shed some light on the nationwide treatment status and outcome of MDR-TB patients in Japan.

**Methods:**

Characteristics and treatment status and outcome of MDR-TB patients notified between 2011 and 2013 were evaluated using the data from the Japan TB Surveillance (JTBS) system. Since the treatment outcome from the surveillance data was not directly linked to any clinical records or drug susceptible test results, we also analyzed the treatment duration of MDR-TB cases in an attempt to validate our results.

**Results:**

Between 2011 and 2013, a total of 172 MDR-TB patients had been notified to the JTBS as MDR-TB. 68.6% (118/172) were males and 70.9% (122/172) were Japan-born – however, over the study period, the proportions of foreign-born, of those in the age group 15–64 years old and of new cases have increased. The overall treatment completion rate was 57.0%, however, when restricted to patients aged 64 years old and below, the rate improved to 71.6%. Treatment duration of 29.2% of those patients who had been recorded as “treatment completed” in fact fell short of the 540 days, the minimum duration as recommended by the Japanese guideline.

**Conclusions:**

Increasing proportion of new cases, and of younger age groups among the MDR-TB patients indicate new transmissions. Better strategies for early detection and containment of MDR-TB are urgently needed. The overall treatment completion rate was 57.0% over the three-year study period. However, when restricting the result to those aged 64 years old and below, the rate improved to 71.6%, which was comparable to similarly industrialized countries. Due to the limitations of the JTBS data, a comprehensive survey of all MDR-TB patients may be necessary to provide more concrete evidence for decision-making.

**Electronic supplementary material:**

The online version of this article (10.1186/s12879-018-3353-9) contains supplementary material, which is available to authorized users.

## Background

Anti-tuberculosis (TB) drug resistance has become one of the major global public health concerns. In 2016 globally, according to the WHO, an estimated 153,119 cases of multidrug resistant-TB (MDR-TB), defined as TB with resistance to both isoniazid (INH) and rifampin (RFP), and rifampicin-monoresistant TB (RR-TB) were detected and notified [1]. MDR-TB imposes higher costs both for the society and the individual patients than drug-susceptible TB, which is associated with longer treatment with more expensive and toxic medications, greater productivity losses and higher mortality and morbidity [2, 3].

Japan is a TB middle-burden country, with a notification rate of 13.9 per 100,000 populations in 2016. MDR-TB is notified to the Japan TB Surveillance system (JTBS), according to which the overall proportion of MDR-TB out of all culture confirmed TB patients was 0.4% in 2016 (50/11151), with the number of notification fluctuating between 50 and 69 over the past decade [4]. The Tuberculosis Research Committee, which is a nationwide coalition of TB hospitals in Japan, has also been conducting nationwide drug resistance surveys approximately every 5 years since 1957. Its most recently published report has similarly indicated the prevalence of MDR-TB to be 0.2% [5]. Despite MDR-TB patients being notified to the JTBS, the treatment outcome has not been evaluated to date. This is because the treatment outcome is currently calculated via a computerized algorithm within the JTBS, which had been designed solely to determine the outcome of pulmonary TB patients receiving the standard regimen – in other words, under the current system, the treatment outcome of all other types of TB not receiving the standard regimen, including extra-pulmonary diseases and MDR-TB, are classified as “unevaluated”. Several hospital-based studies have been conducted to determine the treatment outcome of MDR-TB patients [6–8], and a recent systematic review of treatment outcome in Japan by Mori et al. has established a pooled cure rate of 55% [9]. In this study, we sought to shed some light on the nationwide treatment status and outcome of MDR-TB patients notified in 2011, 2012 and 2013 in Japan, using the data other than that produced by the abovementioned algorithm, from the JTBS.

## Methods

Japan introduced its first electronic surveillance system in 1987, which since then has undergone several major system revisions. Details of the system can be found elsewhere [4]. At the end of each year, it produces four sets of data; 1) a list of all active and latent tuberculosis infection (LTBI) cases newly notified in that year (“newly notified dataset”), 2) a list of all active and LTBI cases registered at the end of the year (“end of year dataset”), 3) a list of active and LTBI cases notified in the previous year, with treatment outcomes (“cohort dataset”), and 4) a list of all cases who were de-registered in that year (“de-registered dataset”). Cases are deregistered when the public health center judges that treatment and/or follow-up is no longer necessary – i.e. when the two-year follow-up following treatment completion, which is mandated by the Infectious Diseases Control Law, is completed, and when the patient has died.

As mentioned, although the “cohort dataset” includes all patients with active TB, including MDR-TB, and LTBI patients notified in the previous year, the treatment outcome is only available for pulmonary TB. Instead of using the treatment outcome from the “cohort dataset”, we thus first extrapolated a list of those MDR-TB patients notified in each year from the “newly notified dataset”, and by linking the list to the subsequent “end of year dataset” via patient ID, referred to the data entered under the field “reasons for terminating the treatment”, and “reasons for de-registering from the system” to determine the treatment status and/or outcome. Treatment outcome and/or status were analyzed by age groups and country of birth, and proportions were compared using chi-squared test or Fisher’s exact test as appropriate.

Since the treatment outcome was not directly linked to any clinical records or drug susceptible test results, we attempted to validate our results by analyzing the treatment duration of MDR-TB patients who had “treatment completed” recorded as their reason for terminating the TB treatment. Since the Japanese guideline states that treatment for MDR-TB patients to continue for a minimum of 18 months after negative conversion, characteristics of those who had completed treatment but whose treatment duration fell short of 540 days were compared with those whose treatment duration was 540 days or longer.

All statistical analysis was conducted using R version 3.1.3 (R Development Core Team, Vienna, Austria).

### Definitions

Patients with MDR-TB: We defined MDR-TB patients as those who, under the non-compulsory data entry field of “drug susceptible test results” in the “newly notified dataset”, were reported as either 1) “resistant to both INH and RFP” in 2011, or 2) “resistant to INH” and “resistant to RFP” after 2012. Prior to 2011, only the combined results of susceptibility tests for INH and RFP were entered, and not the individual test results for each drug.

MDR-TB treatment outcome: We determined that the treatment was completed for those patients whose “reason for terminating the treatment” was recorded as “treatment completed”. Other outcomes included “TB related death”, “non-TB related death”, “transferred out”, “still on treatment”, “treatment stopped due to adverse effects”, “treatment stopped due to reasons other than adverse effects”, “treatment not started”, and “others and unknown”. Transferred out includes both transfer within and to outside Japan, however, the JTBS does not differentiate between the two. Those who have been transferred out within the country and were transferred into another area were tracked via the patient ID – however, the current JTBS does not allow following-up of those who have transferred out of the country. As data entry to the field of “reason for terminating the treatment” is not compulsory, where there was missing data, we also referred to the “reason for de-registering from the system” from the “deregistered dataset”.

Treatment duration: “Treatment duration” is a non-compulsory data entry field and is entered as number of days. Where there was missing data, we referred to the “date when treatment started” and “date when treatment was terminated” to calculate the treatment duration manually.

## Results

### Overview of the MDR-TB patients

Between 2011 and 2013, a total of 172 had been notified to the JTBS as MDR-TB patients. General profile of MDR-TB patients is summarized in Table 1. 68.6% (118/172) were males and 70.9% (122/172) were Japan-born – however, over the three years, both the number and the proportion of foreign-born have increased, from 20.3% (12/59) in 2011 to 32.7% (16/69) in 2013. 55.2% (95/172) and 44.8% (77/172) were in the age groups 15–64 years old and 65 years old and above respectively. There were no patients aged 14 years old and below. The proportion of those in the age group 15–64 years old has increased over the three years, from 54.2% (32/59) in 2011 to 61.2% (30/49) in 2013. 65.1% (112/172) were new patients. Looking at the proportion of new and retreatment cases by country of birth, the increase in the proportion of new cases was observed both among the Japan- and foreign-born patients. However, the increase was larger among the foreign-born (66.7%, 8/12 in 2011, to 75.0%, 12/16 in 2013) compared with the Japan-born patients (60.9%, 28/46 in 2011 to 64.5%, 20/31 in 2013). There were no HIV positive MDR-TB patients notified to the JTBS during the study period.

**Table 1:** General profile of the MDR-TB patients notified between 2011 and 2013

Notification year	2011		2012		2013		2011–2013	
	n	%	n	%	n	%	n	%
Total	59	100.0	64	100.0	49	100.0	172	100.0
Sex								
Male	36	61.0	47	73.4	35	71.4	118	68.6
Female	23	39.0	17	26.6	14	28.6	54	31.4
Country of birth								
Japan-born	46	78.0	45	70.3	31	63.3	122	70.9
Foreign-born	12	20.3	15	23.4	16	32.7	43	25.0
Unknown	1	1.7	4	6.3	2	4.1	7	4.1
Age groups								
0–14	0	0.0	0	0.0	0	0.0	0	0.0
15–64	32	54.2	33	51.6	30	61.2	95	55.2
65+	27	45.8	31	48.4	19	38.8	77	44.8
Treatment history								
New	37	62.7	42	65.6	33	67.3	112	65.1
Retreatment	22	37.3	22	34.4	16	32.7	60	34.9

*MDR-TB* Multidrug-resistant TB, New: patient receiving TB treatment for the first time, Retreatment: patient who has previously received anti-TB drugs for more than 1 month

### Treatment status/outcome

Treatment status and/or outcome for each year cohort and for the entire cohort at the end of 2016 is summarized in Table 2. The overall treatment completion rate was 57.0% (98/172). 5.8% (10/172) had stopped treatment, and 24.4% (42/172) had died, approximately half of whom were due to non-TB related causes. 9.9% (17/172) had transferred out and could not be tracked. At the end of 2016, one patient was still on treatment, and there was no information available for two patients. Two patients, though notified, did not start treatment.

**Table 2:** Treatment outcome of MDR-TB patient notified between 2011 and 2013

Notification year	2011		2012		2013		2011–2013
Evaluation year	End of 2013	End of 2016	End of 2014	End of 2016	End of 2015	End of 2016	End of 2016
Total (%)	59 (100.0)	59 (100.0)	64 (100.0)	64 (100.0)	49 (100.0)	49 (100.0)	172 (100.0)
Treatment completed	32 (54.2)	35 (59.3)	30 (46.9)	33 (51.6)	25 (51.0)	30 (61.2)	98 (57.0)
Treatment stopped (adverse effects)	4 (6.8)	4 (6.8)	2 (3.1)	2 (3.1)	2 (4.1)	2 (4.1)	8 (4.7)
Treatment stopped (other reasons)	1 (1.7)	1 (1.7)	1 (1.6)	1 (1.6)	0 (0.0)	0 (0.0)	2 (1.2)
Died (TB)	9 (15.3)	9 (15.3)	7 (10.9)	8 (12.5)	5 (10.2)	5 (10.2)	22 (12.8)
Died (non-TB)	5 (8.5)	5 (8.5)	10 (15.6)	10 (15.6)	5 (10.2)	5 (10.2)	20 (11.6)
Still on treatment	4 (6.8)	0 (0.0)	6 (9.4)	1 (1.6)	5 (10.2)	0 (0.0)	1 (0.6)
Transferred out	4 (6.8)	4 (6.8)	5 (7.8)	6 (9.4)	7 (14.3)	7 (14.3)	17 (9.9)
Unknown	0 (0.0)	1 (1.7)	1 (1.6)	1 (1.6)	0 (0.0)	0 (0.0)	2 (1.2)
Treatment not started	0 (0.0)	0 (0.0)	2 (3.1)	2 (3.1)	0 (0.0)	0 (0.0)	2 (1.2)

Treatment completion rate increased for each cohort by the end 2016 – this was largely attributable to those who had still been on treatment at the end of 24 months completing treatment by the end of 2016.

Tables 3 and 4 indicate the treatment outcome of the entire cohort at the end of 2016 by age group and country of birth, respectively. The proportion of those who had completed treatment was significantly higher among the age group 15–64 years old compared with those aged 65 years and above (71.6% vs 39.0%, *p* < 0.001). The proportion of those who had died, due both to TB and non-TB causes, was significantly higher among the age group 65 years old and above (4.2% vs 49.4%, *p* < 0.05).

**Table 3:** Treatment outcome at the end of 2016, by age group (*n* = 172)

	15–64 years old		65+ years old	
	n	%	n	%
Total	95	100.0	77	100.0
Treatment completed	68	71.6	30	39.0
Treatment stopped^✝^	4	4.2	6	7.8
Died^✜^	4	4.2	38	49.4
Still on treatment	1	1.1	0	0.0
Transferred out	17	17.9	0	0.0
Unknown	0	0.0	2	2.6
Treatment not started	1	1.1	1	1.3

^✝^Treatment stopped due to adverse effects and other reasons combined, ^✜^ Died due to TB and non-TB causes combined

**Table 4:** Treatment outcome at the end of 2016, by country of birth (*n* = 165※)

	Japan-born		Foreign-born^※^	
	n	%	n	%
Total	122	100.0	43	100.0
Treatment completed	71	58.2	24	55.8
Treatment stopped^✝^	10	8.2	0	0.0
Died^✜^	38	31.1	1	2.3
Still on treatment	0	0.0	1	2.3
Transferred out^☨^	0	0.0	17	39.5
Unknown	2	1.6	0	0.0
Treatment not started	1	0.8	0	0.0

^※^Excludes those whose country of birth is unknown, ^✝^Treatment stopped due to adverse effects and other reasons combined, ^✜^ Died due to TB and non-TB causes combined, ^☨^ Include those who have moved out of Japan

Looking at the treatment outcome by country of birth, the proportion of those who had completed treatment was similar, yet the proportion of those who had died was significantly higher among the Japan-born patients (31.1% vs 2.3%, *p* < 0.05). All those who had transferred out of the entire cohort were foreign-born patients.

#### Treatment duration of those who have “completed treatment”

**Fig. 1:**
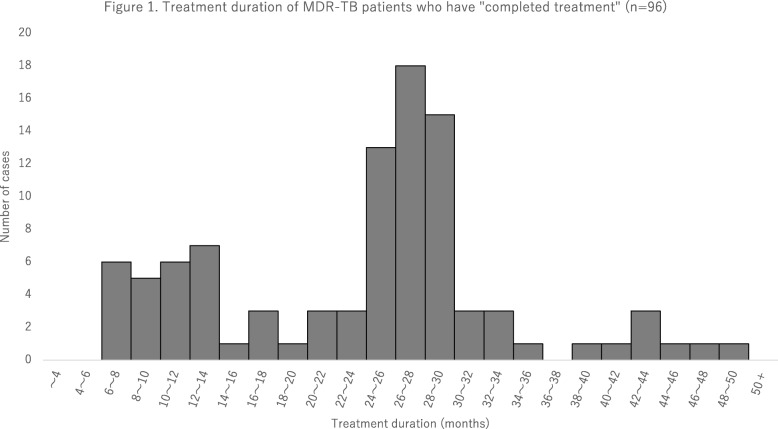
Treatment duration of MDR-TB patients who have “completed treatment” (*n* = 96)

Information regarding treatment duration was available for 96 out of 98 patients who had completed treatment. The mean treatment duration was 713.2 days and the median, 782.0 days (interquartile range; 459.0–857.5 days). Fig. 1 shows the histogram of treatment duration in months – there was a large peak of those completing treatment at around 24 to 30 months (see also Additional file 1). However, there was another smaller peak of those apparently completing treatment at around 12 months. Of the 96 patients, treatment duration fell short of 540 days in 29.2% (28/96). When basic characteristics of those who have completed treatment with treatment duration of 540 days or longer were compared with those who received treatment of insufficient duration, the proportion of those aged 65 years old and above was significantly higher in the latter (60.7% vs 20.6%, *p* < 0.05) (see Additional file 2).

## Discussion

Our study is the first attempt at capturing the overview of MDR-TB patients including treatment status and/or outcome at a national level, using the data from the JTBS. Though drug resistance surveys have been conducted as mentioned earlier, their main focus has been on the prevalence of each type of drug-resistance of anti-TB drugs and has paid little attention to the characteristics of MDR-TB patients or treatment outcomes [4, 9]. Another study, which has used the data from JTBS, reported on the background of drug-resistant TB notified between 2007 and 2009 in Japan – the proportion of the foreign-born patients has increased significantly since then, from 14.9% (23/154) [10] to 25.0%, as in our results. The increase was also consistently observed over the three years of our study period. The increase in the proportion of the younger age group is largely attributable to the increase in the foreign-born patients, who are mainly in their twenties and thirties, and the similar increase has been observed among the drug-susceptible patients [4]. The burden of MDR-TB among the elderly may appear high compared to other similarly industrialized countries - however, this is not surprising considering that two-thirds of the newly notified cases in Japan are diagnosed among those aged 65 years and above. It is most probable that majority of elderly patients with MDR-TB has acquired resistance via inappropriate and/or incomplete treatment in the past.

The proportion of new cases also showed an increase, from 62.7% in 2011 to 67.3% in 2013. In the above-mentioned study, the proportion of new cases over the period of 2007–2009 was reported to be 55.9% (85/152) [10]. This, along with the increase in the proportion of younger age group, indicates that while cases of previously acquired drug-resistance, which are more likely to be common among the older age group as mentioned above, are decreasing, new transmissions are continuing to occur both among the Japan- and foreign-born population. Our results are in line with another study conducted in Japan, which analyzed the transmission dynamics of MDR-TB and extensively drug-resistant TB (XDR-TB). The authors concluded that the MDR-TB and XDR-TB cases were more frequent among younger than older patients, and among foreign-born than Japan-born patients [11].

There were no cases of MDR-TB diagnosed among those aged between 0 and 14 years old, and those with HIV infection, during the study period. As for children, the results are reasonable, considering the very small and declining number of TB cases diagnosed among this age group – 84 in 2011, 63 in 2012 and 66 in 2013, and the majority probably being new cases [4]. Furthermore, as the proportion of bacteriologically confirmed cases among this age group is small compared to older age groups [4], even smaller number of cases would have undergone drug-susceptibility tests. Thus, assuming the same proportion of those with MDR-TB among the culture confirmed cases i.e. 0.4%, a simple calculation would give us less than one case of MDR-TB among those aged between 0 and 14. As for people living with HIV and AIDS, again, the very prevalence of HIV among the general population has constantly been below 1 per 100,000 population. The most recent data indicates that the prevalence of HIV was 0.80, and of AIDS 0.34, per 100,000 population in 2016 [12]. It should on the other hand be noted that the proportion of those receiving HIV testing among TB patients is very minimal. In 2016, HIV test results were known for only 9.1% of the newly notified TB cases [4], thus there may certainly be underreporting of HIV among MDR-TB cases as well.

As for treatment outcome, the treatment completion rate of 57.0% was similar to that reported in the aforementioned systematic review of the Japanese literatures [8]. The treatment completion rate has been reported to be around 70% in the similarly industrialized countries such as UK and US [13, 14] – however, the apparently low completion rate in Japan is due largely to the large proportion of those aged 65 years old and above who tend to die among our patients. As was shown in the Table 3, the rate improved to 71.6% for those aged below 64 years old. Age may certainly be one of the factors affecting treatment outcome – however, as other variables, which have previously been identified as being significant in determining treatment outcome such as number and type of drugs used and various comorbidities [15–17] were not available from the JTBS, we were not able to conduct a meaningful analysis of risk factors for unfavorable outcomes.

Two issues, potentially with significant policy implications, were identified. The first issue relates to the high rate of transfer out among the foreign-born patients. As had been pointed out in several previous studies, Japan does not currently have a system of cross-national referral service [18–20]. Considering that the country of birth of foreign-born patients in Japan is concentrated to a limited number of countries in Asia, and also that the overall number of foreign-born TB patients is increasing, serious discussions ought to start towards establishing such a cross-country referral mechanism for TB patient.

The second issue relates to those MDR-TB patients whose treatment duration, despite having been recorded as “treatment completed”, falls short of the minimum recommended 18 months. These patients may in fact represent those terminating the treatment due to adverse events, or for other reasons. The proportion of those with insufficient treatment duration was higher among the elderly patients; if these are to be re-classified as “treatment stopped”, then the treatment outcome of those aged 65 years and above may present a very different outlook. A more detailed survey on the background of MDR-TB patients is necessary to investigate the possible reasons why certain patients are terminating their treatment early, despite their record indicating treatment completion.

### Limitations

Our study acknowledges a number of limitations. Firstly, as mentioned under the methods section, our definition of MDR-TB is solely dependent on the data as entered by the staff of public health centers, based on the information they have retrieved from physicians. However, the proportion of MDR-TB out of all culture-confirmed TB as reported from the surveillance data has been relatively consistent with the aforementioned hospital-based drug-resistant surveys. The authors therefore believe that it is justifiable to assume reasonable accuracy in the data entry to JTBS. A second, and perhaps a more serious, limitation is the accuracy in the definition of various treatment status/outcomes. The partial validation by treatment duration has indicated that slightly less than a third of the patients who had apparently completed treatment, did not actually receive the regimen as recommended by the Japanese national guideline. A detailed case study should follow to investigate the potential reasons. Thirdly, the relatively small number of MDR-TB cases has required us to interpret some of our sub-analyses, especially those related to treatment outcomes, with caution. Finally, as the JTBS only collects results of drug susceptibility test results of the four first-line drugs e.g. INH, RFP, streptomycin (SM) and ethambutol (EB), we could not separately identify extensively drug-resistant TB (XDR-TB) from our MDR-TB patients. A high proportion of XDR-TB among MDR-TB has previously been reported in Japan [10, 20], and a recent meta-analysis of XDR-TB cohorts demonstrated treatment success in only 44% of patients, with mortality in the range of 14% to 27% [21]. It is therefore likely that a proportion of MDR-TB patients in our study were XDR-TB, with the latter negatively affecting both treatment duration and outcome. As there is currently no national laboratory database, a study to examine the patient record of all MDR-TB cases, which are kept by local public health centers could reveal a more detailed information on the types of drug-resistance.

## Conclusions

Our study evaluated the characteristics and treatment outcome and/or status of MDR-TB patients notified to the JTBS between 2011 and 2013. The increase in the proportion of new cases among the MDR-TB indicates an urgent need for a better strategy for early detection and containment of MDR-TB. The overall treatment completion rate was 57.0% over the three-year study period. However, when restricting the result to those aged 65 years old and younger, the rate improved to 71.6%, which was comparable to similarly industrialized countries. Due to the limitations of the JTBS data, a comprehensive survey of all MDR-TB patients may be necessary to provide more concrete evidence for decision-making.

## Additional files

Additional file 1: Treatment duration in days of MDR-TB patients who have completed treatment, a table showing the treatment duration in days of each of the 96 MDR-TB patients, whose treatment duration was known. (XLSX 9 kb)

Additional file 2: Characteristics of MDR-TB patients who have completed treatment, by treatment duration, a two-by-two table presenting the demographic characteristics of MDR-TB patients who have completed treatment, by treatment duration i.e. < 540 days and > =540 days. (XLSX 10 kb)
